# Latinx bullying and depression in children and youth: a systematic review

**DOI:** 10.1186/s13643-020-01383-w

**Published:** 2020-06-03

**Authors:** Karen Lutrick, Robert Clark, Velia Leybas Nuño, Sheri Bauman, Scott Carvajal

**Affiliations:** 1 College of Medicine - Tucson, University of Arizona, Tucson, AZ USA; 2 Mel and Enid Zuckerman College of Public Health, Health Promotion Sciences, University of Arizona, PO Box 245016, Tucson, AZ 85724 USA; 3 College of Education, Disability Psychoeducation Studies, University of Arizona, Tucson, Arizona USA

**Keywords:** Bullying, Depression, Adolescent health, Latinx/Hispanic

## Abstract

**Background:**

Bullying is associated with negative health outcomes such as depression. Most studies target non-Latinxs, though they often experience higher rates of bullying and depression. This review examines the inclusion of Latinxs in studies of bullying and depression and factors unique to them.

**Methods:**

Databases were searched for articles related to *bullying* and *depression*. Two reviewers found 957 publications and identified 17 for inclusion.

**Results:**

All 17 studies demonstrated a relationship between bullying and depression. Nine examined variables unique to Latinxs.

**Conclusions:**

Studies that included variables unique to Latinxs found a stronger relationship between bullying and depression. Inclusive measures and design are key to understanding and reducing the consequences of bullying in this population.

## Introduction

The reported prevalence of bullying victimization varies substantially. Most prevalence rates fall between 20 and 60% of adolescents report experiencing bullying victimization within the last year [1–5]. Bullying is a risk factor for depression and suicidality and has other potential negative health effects such as increased drug and alcohol abuse, negative school performance, and increased antisocial behaviors [6–12]. The majority of bullying literature focuses on non-Latinx White adolescents. If non-White adolescents are included, African American adolescents are the most likely population studied. However, Latinx adolescents experience bullying and depression at rates that are often higher than their non-Latinx peers [13–17] and experience victimization attributed to language, perceived citizenship/belonging, and appearance [18, 19]. This distinguishes their experiences from the most common groups studied [20]. This systematic review examines the inclusion of Latinx participants in studies on bullying and depression to identify potential relationships or factors that are unique to this population.

### Bullying and depression

Bullying victimization is sometimes called peer victimization. For the purposes of this review, we will treat them as equal experiences and use the term bullying for simplicity. Bullying requires a power imbalance and can be categorized into several different forms. Most common classifications of bullying are direct or overt (physical, verbal), indirect (relational), and cyber [6, 21, 22]. Bullying is often related to negative physical and psychological health [1, 6, 7, 9, 11, 23, 24]. Bullying research has found different rates of victimization based on race/ethnicity [13–17, 25]. The most recent Centers for Disease Control (CDC) Youth Risk Behavior Survey (YRBS) reported that Latinas experienced higher rates reported higher rates of sad/hopelessness at 46.7% compared to 39.8% of all females and 35.3% of all Latinx youth. While the YRBS does not assume causality, there is preliminary evidence that there is a relationship [26].

The relationship between bullying victimization and depression is well documented, but not fully understood. While the mechanism is not yet understood, a large number of mediators and moderators have been studied in an attempt to better understand the relationship between peer victimization and depression. Gender, race/ethnicity, and age or grade are commonly evaluated when attempting to understand the relationship. Intrapersonal behaviors such as internalizing, likelihood for self-blame, and coping strategy, are sometimes included. Interpersonal attributes such as family relationship, acculturation, peer connectedness, and prosocial behavior are often considered. Additionally, environmental factors have been identified as mediating the relationship, such as school environment, racial/ethnic composition, popularity, and policies.

Mediators related to race/ethnicity are of particular interest in this review because of the population of interest. A case has been made by several scholars to include race/ethnicity in the exploration of the experiences of victims [27–29] in lieu of utilizing it solely as a control variable in analysis. Researchers that include it as a variable of interest found significant results that range from variation in prevalence of victimization and depression to identifying it as a “central context variable” [27].

Of particular interest is the role of acculturation because of the population of interest’s familial history with immigration and acculturation stress. The Latinx community in the USA experiences varying levels of acculturation, influenced by how many generations they have been in the USA, where they live, and familial, cultural, and religious values. For adolescents, acculturation stress within families and peer groups can cause inter- and intra-generational conflict that can leave adolescents vulnerable to both victimization and depression.

### Measuring bullying

The CDC defines bullying as repeated, unwanted aggressive behaviors by peers (non-sibling, non-dating), involving an observed or perceived power imbalance [30]. There are experiences of conflict or relational discomfort that may feel like bullying for the victim that do not meet this definition. Additionally, both bullying and depression are complex and socially embedded constructs. For example, coping theories often include a period of appraisal where the victim evaluates the stressor [31]. When an adolescent is bullied because of something they cannot change, like their race or ethnicity, they are more likely to respond to that stressor with an emotion-based response like sadness [32]. Ignoring the complexity of conflict and these constructs when attempting to understand the relationship between peer victimization and health outcomes is potentially problematic.

### Previous reviews

There are no systematic reviews that focus on bullying within specific race/ethnic groups in the USA, to our knowledge. A few systematic reviews explore bullying prevalence or its health impacts. Selkie, Fales, and Moreno conducted a systematic review on the prevalence of cyber bullying in US middle school students [33]. They found a wide variation in the range of victimization prevalence (3–72%) and inconsistency in the quality of measures used and reported outcomes [33]. Patton and colleagues recently published a review of research strategies in bullying studies [34]. They found 24 original research studies that utilized qualitative strategies in understanding the experiences of bullies and victims [34]. Two reviews explored the efficacy of bullying prevention interventions [35, 36]. Both reviews conclude that in general, bullying interventions were effective, with curriculum focused on changing the behavior of aggressors being the least effective strategy. These reviews also noted an inconsistent relationship of reduction in bullying to a reduction in bullying-linked health outcomes [36].

### Objectives

To conduct a systematic literature review to understand bullying and depression research published within the last 20 years that include a Latinx population of at least 25%. Second, to understand if and how race/ethnicity was included in the hypotheses tested, analysis (e.g., sub-group analysis or tests of race/ethnicity as a moderator), results, or discussion.

## Methods

This review followed the Preferred Reporting Items for Systematic reviews and Meta-Analyses (PRISMA) guidelines to the fullest degree possible [37]. The research team included two individuals: a lead researcher and a second reviewer. The second reviewer peer-reviewed the search strategy and participated meaningfully in the selection of articles and identification of data elements for extraction. The research team developed a review protocol that is available by request.

Inclusion criteria included bullying or victimization as a predictor, depression as an outcome (used for brevity and consistency with the literature, though the studies are measuring depressive symptoms outside of a clinical context and not providing a depression diagnosis), Latinx population of at least 25%, US-based, participants younger than 26 years old, empirical research that examined the direct relationship between bullying and depression, and study design that included etiology, measurement, and/or association. Latinx population of at least 25% was selected because that is representative of the national Latinx population under the adolescent and young adult population in the USA [38]. Exclusion criteria included partner or family aggression, study design that included interventions or program evaluation, and examination of childhood victimization rates on participants over the age of 26 (i.e., the examination of the relationship if depression as an adult and victimization as a child).

The relationship between bullying and depression is included in a wide variety of disciplines, therefore medical, psychology, education, and social science databases were searched as of 1 June 2017. Databases included MEDLINE, PsycINFO, PsycArticles, EBSCO Education, Anthropology Plus, Chicano Database, and Cochrane. Published and grey literature were included in the search in order to be as inclusive as possible.

We used a variety of search terms for bullying (i.e., peer victimization, cyberbullying) and depression (i.e., depressive symptoms, mental health) in both keyword and text searches to increase the likelihood of accurate results. Subject and keywords were utilized as specified by the databases. The search strategy was piloted and refined to ensure accuracy. The following MEDLINE search was the primary search strategy, adjusted for each additional database to match language conventions and keywords:

(“bullying”[MeSH Terms] OR “bullying”[All Fields] OR “peer victimization”[All Fields]) AND (“depressive disorder”[MeSH Terms] OR (“depressive”[All Fields] AND “disorder”[All Fields]) OR “depressive disorder”[All Fields] OR “depression”[All Fields] OR “depression”[MeSH Terms])

Primary inclusion criteria was participant population of at least 25% Latinx. Articles were screened for inclusion in several stages. First, a title screen was conducted by both researchers to ensure each article met the basic inclusion criteria of focusing on the relationship between bullying and depression, suicide, or anxiety and was non-workplace focused. Any conflicts between the researchers with regard to study inclusion were discussed using the detailed inclusion and exclusion criteria identified. If additional inclusion and exclusion criteria were added based on the resolution of the dispute, the articles were rescreened to ensure adherence to the revised criteria. An example of that revision was the inclusion of studies exploring the relationship between bullying and suicide. They were included in the initial screening and included in the final selection if they included depression as one of the outcomes of interest.

The second screen was conducted by one researcher and exclusively eliminated studies with a participant population of less than 25% Latinx. Because the participant demographics were presented in a variety of sections and sub-sections throughout the articles, this stage included a full-text screening.

The final screening included a second full-text screen of the articles to ensure relevance and adherence to the inclusion and exclusion criteria. A thorough review and recommendation for inclusion/exclusion was conducted by one researcher and presented to a second researcher for review. The reviewers agreed on all but one article. The disagreement was resolved through discussion and detailed review of the objectives of the review inclusion/exclusion criteria.

Data extraction included information about the participant population, study design, outcomes of interest, results, analysis plan, conclusions, limitations, and bias. If the study was a secondary data analysis of a national study, sample size, population, location, and measures were compared among the final selected studies to reduce the likelihood of multiple reports from the same study. The only variables added after the review started were more specific results fields. We did this to accurately collect the results from the wide variety of analytical strategies employed. The reviewers evaluated each study as low-risk, high-risk, or unclear based upon Cochrane bias assessment with special consideration for confounding criteria because most identified studies were non-interventional. Researchers resolved disagreement utilizing the same mechanism as article screening.

## Results

### Study selection

**Fig. 1:**
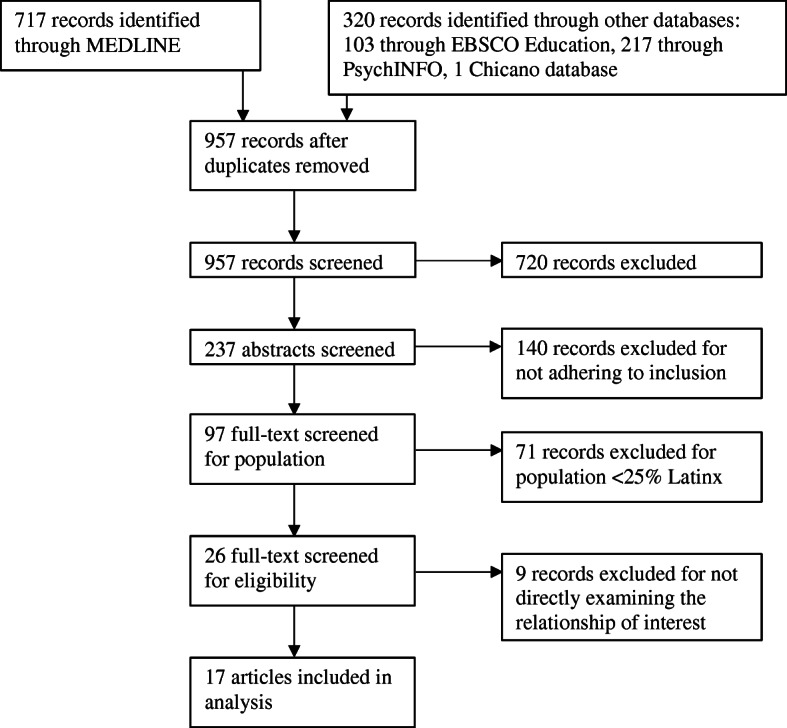
Systematic review selection flow chart

Database searches and hand-searching yielded 1037 articles (see Fig. 1). Once duplicates were removed, 957 articles were screened via title to eliminate articles that were focused on workforce or beyond late adolescent adult populations, family or sibling aggression, a non-US. population or other obvious exclusion criteria. After this screening, 237 were included in an abstract screening, which eliminated all but 96 articles. The final screen for population of interest revealed 26 potential articles, and the final full-text screen for inclusion yielded 17 articles.

The nine articles that were eliminated during the full-text screen were identified as not applicable to the current systematic review because their examination of the relationship between bullying and depression was not directly addressed as a stated outcome or in the analysis plan. They were included in the final stage of screening because of the potential based on the title, abstract, and population and because the researchers did not want to erroneously eliminate articles before a thorough analysis.

### Study characteristics

Descriptive characteristics of the 17 selected studies are included in Tables 1 and 2. It was anticipated that the final study pool was going to be small, so the search results were not limited by date. Ten of the studies were published in the last 5 years and 5 were published before 2010. Fifteen of the studies are peer-reviewed publications and 2 are dissertations, both from the University of Miami. No studies were excluded because the risk of bias was high.

**Table 1:** Descriptions of the selected studies

Citation	Sample	% Latinx	Measure language	Bullying measure	Depression measure	Inclusion of race/ethnicity in analysis
Bauman, 2008 [13]	Elem., *n* = 118	92%	English, Spanish	SEQ-SR	CDI	Yes
Bauman et al 2013 [13]	High, *n* = 1491	40%	English	YRBSS	YRBSS	Yes
Bauman and Summers, 2009 [22]	Middle, *n* = 229	100%	English, Spanish	SEQ-SR	CES-DC	Yes
Bogart et al. 2014 [14]	Elem., middle, high *n* = 4297	44%	English, Spanish	PEQ	DIS-CPS	Control
Cardoso et al. 2017 [44]	Middle, high *n* = 594	100%	English	CA Kids*	CES-DC	Yes
Forster et al. 2013 [26]	High *n* = 1167	88%	English	CA Kids	CES-D	Yes
Garnett et al. 2014 [2014]	High *n* = 965	29%	English	BYS	MDS	Yes
Harrison, 2006 [39]	High *n* = 413	79%	English	R-PEQ	Beck	Yes
Landoll et al. 2013 [15]	High, college *n* = 322	25%	English	R-PEQ	CES-D	Control
Landoll et al, 2015 [40]	High *n* = 839	73%	English	R-PEQ	CES-D	Control
Mihalas, 2008 [2008]	Middle *n* = 153	53%	English, Spanish	SEQ-SR	CDI	Yes
Reed et al. 2015 [43]	High *n* = 15425	30%	English	YRBSS	YRBSS	No
Romero et al. 2013 [2013]	High *n* = 650	100%	English	YRBSS	YRBSS	No
Saluja et al. 2004 [46]	Middle, high *n* = 9863	48%	English	Own	DSM*	Yes
Schacter and Juvonen, 2017 [47]	Middle *n* = 5374	31%	English	Own	CES-D	Control
Storch et al. 2005	Elem. *n* = 186	78%	English	SEQ-SR	CDI	Yes
Wang et al. 2011 [48]	Middle *n* = 7313	26%	English	Olweus	CES-D	Control

Notes: ^a^ modified existing measure; *Abbreviations*: *PEQ* Peer Experience Questionnaire original and revised versions, *SEQ-SR* Social Experience Questionnaire–Self-Report, *YRBS* CDC Youth Risk Behavior Survey, *CA Kids* California Health Kids Survey, *BYS* Boston Youth Survey, *ORB* Olweus Revised Bully/Victim Instrument, *C-PEQ* Cyber-Peer Experiences Questionnaire, *SN-PEQ* Social Networking-Peer Experiences Questionnaire, *CES-D* Center for Epidemiological Studies Depression Scale for Children, *CDI* Children’s Depression Inventory–Short Form, *MDS* Modified Depression Scale, *DSM* Diagnostic and Statistical Manual of Mental Disorders

**Table 2:** Characteristics of the selected studies

Design and study population	
Sample size (*n*) median (range)	2,905 (118–15,425)
> 50% of participants Latinx	9 studies
Participants in high school	12 studies
Studies that include a follow-up period	5 studies
Bullying measures	
R-PEQ/PEQ	4 studies
SEQ-SR	4 studies
YRBS	3 studies
Olweus	1 study
Other measures (own, modified, regional, etc.)	9 studies
Depression measures	
CES-D	5 studies
YRBS	3 studies
CDI	3 studies
CES-DC	2 studies
Bullying types measured	
Relational	8 studies
Physical/overt	8 studies
Cyber	6 studies
No subtypes measured	4 studies

*Abbreviations*: *PEQ* Peer Experience Questionnaire original and revised versions, *SEQ-SR* Social Experience Questionnaire–Self-Report, CDC Youth Risk Behavior Survey, *CES-D* Center for Epidemiological Studies Depression Scale for Children, *CDI* Children’s Depression Inventory–Short Form

### Participant overview

The studies included a total population of 49,399 (range 118–15,425) ranging from elementary school through high school. Twelve included high school, 7 included middle school, 3 included elementary school, and 1 included college, with 5 of the studies including more than 1 age group (Table 1). The mean percentage of Latinx participants was 61% (range 25–100%) and the mean percentage of female participants was 58% (range 49–100%). Three studies had samples that were 100% Latinx participants, including one study that was also 100% female.

### Measures overview

The selected studies utilized a variety of victimization measures (Table 1 and Table 2). Two instruments were used by four studies each: the Peer Experience Questionnaire original and revised versions (PEQ) [14, 15, 39, 40] and the Social Experience Questionnaire–Self-Report (SEQ-SR) [13, 16, 22, 41], three studies utilized the CDC Youth Risk Behavior Survey (YRBS) questions regarding bullying behavior [17, 42, 43], two utilized the California Health Kids Survey [26, 44], one study used the Boston Youth Survey [45], one study used the Olweus Revised Bully/Victim Instrument (ORB) (46), and the remaining two utilized their own measure [46, 47]. In addition to a more traditional bullying measure, two studies also added a measure attempting to identify unique outcomes related to cyber bullying utilizing the Cyber-Peer Experiences Questionnaire (C-PEQ) [40] and Social Networking-Peer Experiences Questionnaire (SN-PEQ) [15] measures.

For depression measures, seven studies utilized the Center for Epidemiological Studies Depression Scale for Children (CES-D or CES-DC) [15, 22, 26, 40, 44, 47, 48], three studies utilized the questions in the YRBS [17, 42, 43], and four studies utilized the Children’s Depression Inventory–Short Form (CDI) [13, 16, 41].

In addition to the victimization and depression symptom measures, several studies utilized measures intended to measure mediators, moderators, and protective factors. To understand the role of acculturation on the relationship between bullying and depression, the Brief Acculturation Rating Scale for Mexican Americans II (ARMSA-II) [13, 22, 26], Family Adaptability and Cohesion Evaluation Scale (FACES-II) [26], and Acculturative Stress Scale (modified) were utilized [26]. Other potential mediators and moderators were examined utilizing the Child and Adolescent Social Support Scale (CASSS) [16], Spiritual Assessment Instrument (SSA) [16], and Children’s Hope Scale (CHS) [16], Multidimensional Scale of Perceived Social Support [26], and Self-Perception Profile [14]. Measures utilized to study outcomes other than depression were Pediatric Quality of Life Inventory [14], Social Anxiety Scale for Adolescents (SAS-A) [15], Social Anxiety Scale for Children–Revised (SASC-R) [41], and Asher Loneliness Scale (ALS) [41]. Suicide risk and substance abuse questions in the YRBS were incorporated in the analysis [26, 45].

Only one study included a qualitative component in the form of interviews [16].

## Discussion

### Summary of evidence

#### Main findings

The present systematic review identified a statistically significant relationship between bullying and depression in all studies. Most of the studies treated bullying as the explanatory variable and depression/depressive symptoms as the outcome (see Table 3). The only exception was Schacter and Juvonen [47]. They identified that depression led to behaviors which increased the risk of perceiving peer victimization through a prospective, longitudinal study [47].

**Table 3:** Summary of results

Citation	Bullying types	Results	Analysis method	Conclusion
Bauman, 2008 [13]	Relational, overt	Coefficient, *p* value: *β* = 0.32, *p* < 0.009 (relational) *β* = 0.09, *p* = 0.396 (overt)^a^	Regression	Relational victimization had the strongest, and only significant, relationship with depression
Bauman et al. 2013 [13]	Traditional, cyber	Standardized coefficient, *p* value: 0.13, *p* < 0.01 (F, traditional) 0.20, *p* < 0.001 (M, traditional) 0.24, *p* < 0.001 (F, cyber) 0.10, *p* = 0.10 (M, cyber)^a^	SEM	Depression was a mediator for the relationship between traditional bullying and suicide for female and male participants, but only for female in cyber bullying
Bauman and Summers, 2009 [22]	Relational, overt	Coefficient, *p* value: *β* = 0.30, *p* < 0.000 (relational) *β* = 0.29, *p* < 0.000 (overt)	Regression	Victimization significantly predicted depression
Bogart et al. 2014 [14]	No distinction	Coefficient, *p* value: *β* = 0.12, *p* < 0.001 (present) *β* = 0.43, *p* < 0.001 (past) *β* = 0.79, *p* < 0.001 (past and present) For 10th grade, versus non-victims	Regression	Experiencing present victimization with a history of past victimization related to the strongest relationship with depression
Cardoso et al, 2017 [44]	Verbal, Physical, Ethnic-Biased	Unstandardized coefficient, *p* value: 0.585, *p* < 0.05 (relational) 0.413, *p* < 0.05 (ethnic-biased) NR, *p* = NR (physical)^a^	SEM	Relational and ethnic-biased victimization were significantly associated with depression, but physical bullying was not
Forster et al. 2013 [26]	Direct, indirect	Coefficient, *p* value: *β* = 0.25, *p* < 0.0001	Regression	Peer victimization, acculturative stress and lower family cohesion were risk factors for depression
Garnett et al. 2014 [45]	No distinction	Coefficient, *p* value: *β* = 2.84, *p* < 0.01 (bully × disc)	LCA, regression	The intersection of discrimination and bullying victimization was associated with depression
Harrison, 2006 [39]	Overt, relational, reputational	Coefficient, *p* value; time 1: *β* = 0.16, *p* < 0.01 (overt) *β* = 0.19, *p* < 0.01 (relational) *β* = 0.07, *p* = NR (reputational)^a^ Coefficient, *p* value; time 2: *β* = 0.06, *p* = NR (overt)^a^ *β* = − 0.12, *p* < 0.05 (relational) *β* = 0.19, *p* < 0.01 (reputational)	Regression	Peer victimization was generally associated with high depression, but causal and moderation patters differed based on type of victimization.
Landoll et al, 2013 [13]	Relational, overt, cyber	Standardized coefficient, *p* value: 0.40, *p* < 0.01 (cyber) 0.23, *p* = 0.04 (relational)	SEM	Peer victimization was related to higher rates of depression and anxiety with a specific examination of victimization on social media networks
Landoll et al, 2015 [40]	Relational, reputational, overt, cyber	Standardized coefficient, *p* value: NR (overt)^a^ 0.41, *p* < 0.001 (relational) NR (reputational)^a^ 0.16, *p* < 0.05 (cyber)	SEM	Relational and cyber bullying contribute to depression, with cyber bullying having a unique effect
Mihalas, 2008 [16]	Relational, physical, verbal	Coefficient, *p* value: *β* = 0.46, *p* < 0.0001	Regression	Relational victimization was significantly associated with depression; hope and perceived social support were significant moderator variables
Reed et al, 2015 [43]	Traditional, cyber	Unstandardized coefficient, *p* value 0.57, *p* < 0.001 (traditional) 0.58, *p* < 0.001 (cyber)	PME	There were statistically significant paths from victimization to depression and suicide without involvement of depression, suicidal thinking or suicide planning
Romero et al, 2013 [17]	Traditional, cyber	Correlation, *p* value: 0.16, *p* < 0.001 (traditional) 0.19, *p* < 0.01 (cyber)	Correlation	Victimization correlated to depression; being a victim increased the likelihood of suicide after controlling for depression
Saluja et al, 2004 [46]	No distinction	Prevalence, risk ratio, 95% CI: 27.7%, RR 1.2, (1.1–1.6); F, 1–2× 36.8%, RR 1.7, (1.4–2.1); F, 2 + 10.2%, RR 1.4, (0.9–2.1); M, 1–2× 17.7%, RR 2.4, (1.7–3.4); M, 2+	Prevalence	Both bullies and victims were more than twice was likely to report depression
Schacter and Juvonen, 2017 [47]	No distinction	Coefficient, *p* value 0.143, *p* < 0.001 (depression led to bullying)	Mediation model	Depression for the adolescent and friend group increase the risk for perceptions of victimization through a self-blaming attributions model
Storch et al, 2005	Relational, overt	Standardized coefficient, *p* value: 0.56, *p* < 0.001 (overt, boys) 0.47, *p* < 0.001 (overt, girls) 0.23, *p* > 0.05 (relational, boys)* 0.31, *p* > 0.001 (relational, girls)	Linear regression	Overt and relational victimization were positively associated with depressive and other social-psychological adjustment symptoms
Wang et al, 2011 [48]	Physical, verbal, relational, cyber	Prevalence, *R*^2^: 21.2%, *R*^2^ = 0.115 (physical) 53.7%, *R*^2^ = 0.170 (verbal) 51.6%, *R*^2^ = 0.189 (relational) 13.8%, *R*^2^ = 0.107 (cyber)	Prevalence, regression	Depression was associated with all four types of bullying

NOTES: ^a^Relationship not significant; *Abbreviations*: *SEM* Structural Equation Modeling, *LCA* Latent Class Analysis, *PME* Path Model Estimate

Thirteen of the studies examined different forms of peer victimization, such as direct, indirect, or cyber. While they all found an overall relationship between bullying and depression, some found no relationship when specifically examining direct or physical bullying [13, 15, 44]. For some, the relationship between direct bullying and depression was related to gender, with no relationship identified in groups of boys [49]. There is evidence that suggests cyber bullying is unique and distinct from traditional forms of bullying (physical, relational and verbal), often demonstrating a stronger relationship between victimization and depression than traditional bullying [15, 17, 40].

Most of the studies collected data at one time point to identify the relationship of interest. Bogart and colleagues utilized data collected at three time points: grades 5, 7, and 10, and found that depression was more likely to occur for individuals who had experienced peer victimization in the past and even more likely to occur for individuals who had experienced peer victimization in the present and past [14].

Three studies specifically focused on suicide planning, ideation and attempt in addition to depressive symptoms [17, 42, 43]. They all found a relationship between bully victimization and suicide. These three studies all indicate a relationship between victimization and suicide, but the exact pathway and mechanisms underlying the relationship are not fully described nor tested to date. Since Latina adolescents have a higher prevalence of depression and suicide as compared to their peers, this relationship should continue to be explored [3].

#### Latinx-specific factors

Of particular interest for this review is the inclusion of the Latinx population at frequencies that are close to those of the national population. Nine of the selected studies included race/ethnicity as a variable of interest and/or component of the analysis. These studies hypothesized that race/ethnicity interacted with the relationship between peer victimization and depression in some way.

Three of the studies hypothesized that acculturation would interact with the relationship of interest and included the ARMSA-II measure in the study design [13, 22, 26]. The significance of the results varied between the three studies. In her 2008 study, Bauman did not find a relationship between victimization and depression when looking at acculturation in elementary school students [13]. Bauman and Summers found that individuals with scores on the ARMSA-II that indicated more anglo-oriented traits reported more depression when victimized than their bicultural peers [42]. Forster and colleagues examined acculturative stress as well as family cohesion and found that both were significant predictors of depression in peers that experienced victimization [26]. As these results are mixed, additional studies with comparable measures across various Latinx subgroups (based on nation of origin or acculturation factors) may be necessary to more fully identify the conditions in which the relationships are observed. Also, the findings of Foster and colleagues indicate that acculturative stress may be an important consideration when examining the relationship between peer victimization and depression in Latinx (and potentially other immigrant-origin) youth.

Cardoso and colleagues and Garnet and colleagues included race/ethnicity in their studies by considering the interaction and/or overlap of bullying and discrimination for minority adolescents [44, 45]. Cardoso added a type of bullying they call “ethnic-biased” bullying to their analysis. To measure this, they added one question to the bullying survey asking the participant if they felt they were being bullied based on their race, ethnicity or country of origin [44]. They found that both ethnic-biased and relational bullying were significantly associated with depression. They conclude that, like cyber bullying, there may be a differential effect on depression when bullying is perceived as based on ethnic bias. While the results are promising, they measured ethnic-biased bullying with one question that directly asks the victim. This could have led to a less reliable and comprehensive assessment and one where bias due to prompting may be evident. Nonetheless, Cardoso and colleagues introduce a term that deserves the attention of future research. Specifically, a more comprehensive strategy to measure ethnic bias is needed.

Garnett and colleagues (2014) examined the intersection of multiple attributes of discrimination and bullying, utilizing one question about discrimination that was added to the bullying survey [45]. Their definition of discrimination was expanded beyond racial/ethnic to lesbian, gay, bisexual, and transgender (LGBT) and weight-based discrimination. They found that the adolescents experienced bullying based on discrimination strengthened the relationship between victimization and depression [45]. This is consistent with the bias-based literature that has identified a strong relationship between bias-based bullying and negative health outcomes in non-White and sexual minority populations [4, 5, 50, 51].

Two studies included 100% Latinx participants, examining this population exclusively in their analysis [17, 44]. Romero and colleagues (2013) specifically examined teen suicide in the Latina population and the potential relationship with bullying. In addition to finding an increased likelihood of depression and suicide attempts in Latinas that have been victims of bullying, they also found more reports of victimization than other studies with Latinx samples.

### Limitations

Limitations of this systematic review include the inclusion of only studies published in the English language. Since the population of interest is US-based, the likelihood of not including a relevant study is low. Limitations within the studies were consistent and had significant overlap. All of the studies utilized self-report measures, which have the potential to introduce bias. That said, self-reports are the standard in this area, and it is not clear if less subjective measures such as implicit experiences assessments could be developed, let alone employed within large scale surveys. Most of the studies utilized a cross-sectional design, which do not afford empirically based tests of causality. Also, most of the measures were designed and evaluated on a majority population of white students, and thus may miss important nuances of relevance to the diverse Latinx population within the USA. Several of the studies utilized a single item to measure a variable of interest, which does not capture the depth of experience and increases the likelihood of bias [44].

Additionally, several studies noted small sample size as a limitation [13, 16, 22] and one had a comparatively sized sample, but did not note a limitation [41]. This review’s focus on one population limits the generalizability of the results to other populations.

## Conclusion

This systematic review examines the inclusion of Latinx participants in studies on bullying and depression to identify potential relationships or factors that are unique to this population. Of the 17 studies identified, 9 of them included specific factors related to race/ethnicity as variables of interest. Several factors identified suggest that the experiences of Latinx adolescents and other immigrant-based populations may reflect sociocultural factors that are significantly different from those of their white peers. Additional examination of this phenomenon in larger populations and different immigrant populations should be conducted to continue to examine the relationship. Additional consideration of studies that utilize more construct-precise measures, such as clarifying if bullying is discriminatory or bias-based, are important to expanding the knowledge-base on bullying and health. Additionally, designs that are prospective and compare Latinx groups (based on nation of origin and acculturation factors) are critical to advancing the field. Finally, particularly in light of the national discourse around immigration and Mexican-origin persons (the largest sub-group of Latinxs), heterogeneity of samples and efforts to identify new contexts of bullying, such as through qualitative and mixed methods studies, may yield insight helpful to health providers and intervention developers so that the negative downstream consequence of bullying may be more effectively prevented.

The overall Latinx representation in bullying and depression studies is insufficient. The Latinx community is largest ethnic minority group in the USA and in many regions, they comprise an anticipated 26% of the K-12 public school enrollment and is expected to continue to grow faster than other populations [52]. Continued research on the experiences of this population is needed.

## Data Availability

The datasets used and/or analyzed during the current study are available from the corresponding author on reasonable request.

## Abbreviations

PEQ: Peer Experience Questionnaire
YRBS: CDC Youth Risk Behavior Survey
ORB: Olweus Revised Bully/Victim Instrument
C-PEQ: Cyber-Peer Experiences Questionnaire
SN-PEQ: Social Networking-Peer Experiences Questionnaire
CES-D or CES-DC: Center for Epidemiological Studies Depression Scale for Children
CDI: Children’s Depression Inventory–Short Form
ARMSA-II: Brief Acculturation Rating Scale for Mexican Americans II
FACES-II: Family Adaptability and Cohesion Evaluation Scale
CASSS: Child and Adolescent Social Support Scale
SSA: Spiritual Assessment Instrument
CHS: Children’s Hope Scale
SAS-A: Social Anxiety Scale for Adolescents
SASC-R: Social Anxiety Scale for Children–Revised
ALS: Asher Loneliness Scale

## References

[1] Nansel TR, Overpeck M, Pilla RS, Ruan WJ, Simons-Morton B, Scheidt P (2001). Bullying behaviors among US youth: prevalence and association with psychosocial adjustment. J Am Med Assoc.

[2] Modecki KL, Minchin J, Harbaugh AG, Guerra NG, Runions KC (2014). Bullying prevalence across contexts: a meta-analysis measuring cyber and traditional bullying. J Adolesc Health.

[3] Youth Risk Behavior Survey Questionnaire [Internet]. 2017 [cited September 27, 2018]. Available from: www.cdc.gov/yrbs.

[4] Coker TR, Elliott MN, Kanouse DE, Grunbaum JA, Schwebel DC, Gilliland MJ (2009). Perceived racial/ethnic discrimination among fifth-grade students and its association with mental health. Am J Public Health.

[5] Russell ST, Sinclair KO, Poteat VP, Koenig BW (2012). Adolescent health and harassment based on discriminatory bias. Am J Public Health.

[6] Olweus D (2013). School bullying: development and some important challenges. Annu Rev Clin Psychol.

[7] Messias E, Kindrick K, Castro J (2014). School bullying, cyberbullying, or both: correlates of teen suicidality in the 2011 CDC youth risk behavior survey. Compr Psychiatry.

[8] Klomek AB, Kleinman M, Altschuler E, Marrocco F, Amakawa L, Gould MS. Suicidal Adolescents’ Experiences With Bullying Perpetration and Victimization during High School as Risk Factors for Later Depression and Suicidality. The Relationship Between Youth Involvement in Bullying and Suicide. 2013;53(1, Supplement):S37-S42. 10.1016/j.jadohealth.2012.12.008 23790199

[9] Klomek AB, Kleinman M, Altschuler E, Marrocco F, Amakawa L, Gould MS (2011). High school bullying as a risk for later depression and suicidality. Suicide Life Threat Behav.

[10] Garnett B, Masyn K, Austin S, Williams D, Viswanath K (2015). Coping styles of adolescents experiencing multiple forms of discrimination and bullying: evidence from a sample of ethnically diverse urban youth. J School Health.

[11] Brank EM, Hoetger LA, Hazen KP (2012). Bullying. Ann Rev Law Soc Sci.

[12] Wang J, Iannotti RJ, Nansel TR (2009). School bullying among adolescents in the United States: physical, verbal, relational, and cyber. J Adolesc Health.

[13] Bauman S (2008). The association between gender, age, and acculturation, and depression and overt and relational victimization among Mexican American elementary students. J Early Adolesc.

[14] Bogart LM, Elliott MN, Klein DJ, Tortolero SR, Mrug S, Peskin MF (2014). Peer victimization in fifth grade and health in tenth grade. Pediatrics..

[15] Landoll RR, La Greca A, Lai BS. Aversive Peer Experiences on Social Networking Sites: Development of the Social Networking-Peer Experiences Questionnaire (SN-PEQ). Journal of research on adolescence : the official journal of the Society for Research on Adolescence. 2013;23(4). 10.1111/jora.12022 PMC3839674 24288449

[16] Mihalas ST. Positive protective factors as moderators in the relationship between relational victimization and depression in minority adolescents. US: ProQuest Information & Learning; 2008.

[17] Romero AJ, Wiggs CB, Valencia C, Bauman S (2013). Latina teen suicide and bullying. Hisp J Behav Sci.

[18] Hong JS, Kral MJ, Sterzing PR (2015). Pathways from bullying perpetration, victimization, and bully victimization to suicidality among school-aged youth: a review of the potential mediators and a call for further investigation. Trauma Violence Abuse.

[19] Romero AJ, Roberts RE (2003). Stress within a bicultural context for adolescents of Mexican descent. Cultur Divers Ethnic Minor Psychol.

[20] Araújo BY, Borrell LN (2006). Understanding the link between discrimination, mental health outcomes, and life chances among latinos. Hisp J Behav Sci.

[21] Wang J, Iannotti RJ, Luk JW (2012). Patterns of adolescent bullying behaviors: physical, verbal, exclusion, rumor, and cyber. J Sch Psychol.

[22] Bauman S, Summers JJ (2009). Peer victimization and depressive symptoms in Mexican American middle school students: including acculturation as a variable of interest. Hisp J Behav Sci.

[23] Juvonen J, Graham S, Schuster MA (2003). Bullying among young adolescents: the strong, the weak, and the troubled. Pediatrics..

[24] Klomek AB, Kleinman M, Altschuler E, Marrocco F, Amakawa L, Gould MS (2013). Suicidal adolescents’ experiences with bullying perpetration and victimization during high school as risk factors for later depression and suicidality. J Adolesc Health.

[25] Hanish LD, Guerra NG (2000). Predictors of peer victimization among urban youth. Soc Dev.

[26] Forster M, Dyal SR, Baezconde-Garbanati L, Chou C-P, Soto DW, Unger JB (2013). Bullying victimization as a mediator of associations between cultural/familial variables, substance use, and depressive symptoms among Hispanic youth. Ethn Health.

[27] Graham S (2006). Peer victimization in school: exploring the ethnic context. Curr Dir Psychol Sci.

[28] Juvonen J, Nishina A, Graham S (2006). Ethnic diversity and perceptions of safety in urban middle schools. Psychol Sci.

[29] Bucchianeri MM, Gower AL, McMorris BJ, Eisenberg ME (2016). Youth experiences with multiple types of prejudice-based harassment. J Adolesc.

[30] Gladden RM. Bullying surveillance among youths: uniform definitions for public health and recommended data elements. Version 1.0. Centers for Disease Control and Prevention. 2014.

[31] Glanz K, Rimer BK, Viswanath K. Health behavior : theory, research, and practice. Fifth edition. ed. San Francisco, CA: Jossey-Bass; 2015. xxv, 485 pages p.

[32] Folkman S, Lazarus RS (1988). Coping as a mediator of emotion. J Pers Soc Psychol.

[33] Selkie EM, Fales JL, Moreno MA (2016). Cyberbullying prevalence among US middle and high school–aged adolescents: a systematic review and quality assessment. J Adolesc Health.

[34] Patton DU, Hong JS, Patel S, Kral MJ (2017). A systematic review of research strategies used in qualitative studies on school bullying and victimization. Trauma Violence Abuse.

[35] Ttofi MM, Farrington DP (2011). Effectiveness of school-based programs to reduce bullying: a systematic and meta-analytic review. J Exp Criminol.

[36] Vreeman RC, Carroll AE (2007). A systematic review of school-based interventions to prevent bullying. Arch Pediatr Adolesc Med.

[37] Liberati A, Altman DG, Tetzlaff J, Mulrow C, Gotzsche PC, Ioannidis JP (2009). The PRISMA statement for reporting systematic reviews and meta-analyses of studies that evaluate health care interventions: explanation and elaboration. J Clin Epidemiol.

[38] Bureau USC (2013). American community survey, 2010 American community survey 5-year estimates.

[39] Harrison HM (2006). Peer victimization and depressive symptoms in adolescence.

[40] Landoll RR, La Greca AM, Lai BS, Chan SF, Herge WM (2015). Cyber victimization by peers: prospective associations with adolescent social anxiety and depressive symptoms. J Adolesc.

[41] Storch EA, Ledley DR, Lewin AB (2006). Peer victimization in children with obsessive-compulsive disorder: relations with symptoms of psychopathology. J Clin Child Adolesc Psychol.

[42] Bauman S, Toomey RB, Walker JL (2013). Associations among bullying, cyberbullying, and suicide in high school students. J Adolesc.

[43] Reed KP, Nugent W, Cooper RL (2015). Testing a path model of relationships between gender, age, and bullying victimization and violent behavior, substance abuse, depression, suicidal ideation, and suicide attempts in adolescents. Child Youth Serv Rev.

[44] Cardoso JB, Szlyk HS, Goldbach J, Swank P, Zvolensky MJ. General and Ethnic-Biased Bullying Among Latino Students: Exploring Risks of Depression, Suicidal Ideation, and Substance Use. Journal of immigrant and minority health. 2017. 10.1007/s10903-017-0593-5 28493116

[45] Garnett B, Masyn K, Austin S, Miller M, Williams D, Viswanath K (2014). The intersectionality of discrimination attributes and bullying among youth: an applied latent class analysis. J Youth Adolesc.

[46] Saluja G, Iachan R, Scheidt PC, Overpeck MD, Sun W, Giedd JN (2004). Prevalence of and risk factors for depressive symptoms among young adolescents. Arch Pediatr Adolesc Med.

[47] Schacter HL, Juvonen J. Depressive symptoms, friend distress, and self-blame: risk factors for adolescent peer victimization. J Appl Dev Psychol. 2017. 10.1016/j.appdev.2017.02.005 PMC5644397 29056807

[48] Wang J, Nansel TR, Iannotti RJ (2011). Cyber and traditional bullying: differential association with depression. J Adolesc Health.

[49] Storch EA, Nock MK, Masia-Warner C, Barlas ME (2003). Peer victimization and social-psychological adjustment in Hispanic and African-American children. J Child Fam Stud.

[50] Fisher CB, Wallace SA, Fenton RE (2000). Discrimination distress during adolescence. J Youth Adolesc.

[51] Newman PA, Fantus S (2015). A social ecology of bias-based bullying of sexual and gender minority youth: toward a conceptualization of conversion bullying. J Gay Lesbian Soc Serv.

[52] Aud SH, William; Planty, Michael; Snyder, Thomas; Bianco, Kevin; Fox, Mary Ann; Frohlich, Lauren; Kemp, Jana; Drake, Lauren. The Condition of Education 2010. National Center for Education Statistics; 2010.

